# Effects of an external compared to an internal focus of attention on the excitability of fast and slow(er) motor pathways

**DOI:** 10.1038/s41598-021-97168-9

**Published:** 2021-09-09

**Authors:** Yves-Alain Kuhn, Martin Keller, Sven Egger, Wolfgang Taube

**Affiliations:** 1 Department of Neurosciences and Movement Science, Faculty of Science and Medicine, University of Fribourg, Bd. De Pérolles 90, 1700 Fribourg, Switzerland; 2 Department of Sport, Exercise and Health, University of Basel, Basel, Switzerland

**Keywords:** Motor cortex, Attention

## Abstract

The neurophysiological mechanisms underlying the behavioural improvements usually associated with an external (EF) compared with an internal focus of attention (IF) remain poorly investigated. Surround inhibition in the primary cortex has been shown to be more pronounced with an EF, indicating a more spatial restriction of the motor command. However, the influence of different foci on the temporal aspect of the motor command, such as the modulation of fast versus slow(er) motor pathways, remains unknown and was therefore investigated in this study. Fourteen participants were asked to press on a pedal with the right foot to match its position with a target line displayed on a screen. The deviation of the pedal from the target line was used as a behavioural parameter and compared between both foci (EF vs IF). Additionally, conditioned H-reflexes were evoked during the motor task to assess the excitability of fast (direct) and slower (more indirect) motor pathways when adopting an EF or IF. With an EF compared to an IF, the motor performance was enhanced (*P* = .001; + 24%) and the activation of slow(er) motor pathways was reduced (*P* < 0.001, − 11.73%). These findings demonstrate for the first time that using different attentional strategies (EF and IF) has an influence on the excitability of slow(er) motor pathways. Together with the increased intracortical inhibition and surround inhibition known from previous studies, the diminished activation in the slow(er) motor pathways further explains why using an EF is a more economic strategy.

## Introduction

In 1998^1^, the first study investigated the effects of an external focus of attention (EF) compared to an internal focus of attention (IF). An IF is typically induced by directing the attention to the participant’s own body while an EF is defined as directing attention to the effect of the movement on the environment. Based on this initial study, a substantial amount of publications emerged demonstrating performance-enhancing effects with an EF compared to a control condition and/or an IF (see review by Wulf, 2012).

In more recent years, scientists did not only investigate the EF-induced performance gains but also accompanying physiological changes. For example, it has been demonstrated that adopting an EF results in an enhanced performance during a fatiguing task^2^, but also reduced oxygen consumption during running^3,4^. Moreover, studies investigated muscular activation while participants performed identical motor tasks with either an EF or an IF. Most studies showed reduced EMG activity in the agonist^5–10^ and/or the antagonist muscle^9,11^ in the EF condition. These studies support and strengthen the ‘constrained action hypothesis’ that was postulated in 2001^12,13^. In brief, this theory stipulates that adopting an external focus of attention contrasted to an internal one improves motor control and learning by promoting more automatic and unconscious modes of motor control. This theory is not only well supported by previous physiological studies^14,15^ but also on findings from behavioural studies. In this sense, it was demonstrated that an EF decreases attentional capacity demands as subjects produced smaller balance errors and enhanced reaction times when adopting an EF compared to an IF^13^. Furthermore, an EF allowed faster movement adjustments^12,13^ as well as faster reaction times in a dual task paradigm^16^. Despite this good evidence for the constrained action hypothesis, the underlying neurophysiological mechanisms at the cortical level remain rather poorly understood.

So far, it is well known that attention has an impact on activity of the primary motor cortex (M1)^17–19^. However, the first study that aimed to clarify differential brain activation patterns by changing the focus of attention^20^ was conducted with functional magnetic resonance imaging (fMRI). In this study, the impact of motor-related attentional foci (EF versus IF) on brain activity was investigated. The authors reported enhanced brain activity in the primary motor cortex (M1), in primary somatosensory, and insular cortices when participants performed a finger sequence task with an EF. Altered brain activation when switching between attentional foci was also confirmed by another fMRI study from Zimmermann and colleagues^21^. Thus, it seems that attention in general has an impact on the activity of M1 and other brain structures. As a limitation of the initial fMRI studies cited above, it might be argued that those studies were conceptualized to detect between-group differences and thus, different subjects were measured with an EF than with an IF. Furthermore, using intrinsic blood-tissue contrasts^22^ does not allow to differentiate between excitatory and inhibitory neural activity^23^ but provides an estimate about the overall brain activity.

In contrast to fMRI, transcranial magnetic stimulation (TMS) and electroencephalography (EEG) have a high temporal resolution and have also been used to investigate the effect of different attentional foci on neurophysiological parameters. For example, Chow and colleagues^24^ demonstrated that the decreased balance performance (bigger sway path) when using an IF over an EF in young adults was accompanied by a higher T3 (verbal-analytical) and Fz (motor-planning) coherence which reflects increased verbal-analytical involvement during motor planning and control^25,26^. Moreover, using different TMS protocols, recent studies^11,27,28^ demonstrated altered neural activation in M1 for the muscle directly involved in the motor task (prime mover) but also the surrounding and/or antagonistic muscles. For example, in a study with elaborated TMS protocols (paired-pulse and subthreshold stimulation paradigms), Kuhn and co-workers^27^ demonstrated that adopting an EF contrasted to an IF enhances cortical inhibition within M1. The enhanced activity of inhibitory interneurons within M1 may indicate a reduced motor-cortical activation^11,27,28^, which could be interpreted as a more efficient and more automatic mode of motor control when adopting an EF contrasted with an IF.These data might therefore be considered as a more efficient neural strategy. Besides the effects found in the prime-mover, there is also evidence that adjacent muscles are activated differently when participants use an EF versus an IF. With a single-pulse TMS protocol, Kuhn and colleagues^11^ investigated the effects of different attentional strategies on the modulation of surround inhibition (SI). The authors reported not only enhanced force levels (behavioural improvement) but also increased levels of SI in the antagonistic muscle (‘spatial’ neurophysiological change) when adopting an EF. Thus, it might be argued that using an EF allowed the motor-related brain areas to minimize unnecessary contractions of muscles that were not directly involved in the task, what might be a second neural mechanism explaining the EF-induced performance gains and the more economic functioning. These two studies are therefore the first studies that shed some light into the differential activation of brain centres when using an EF compared with an IF. Furthermore, these studies further support the ‘constrained action hypothesis’ cited above by showing a more spatially focused (i.e. more economic) motor command when applying an EF.

Interestingly, in both Kuhn and colleagues’ studies (2016 & 2018), the single-pulse motor evoked potentials (MEPs) in the muscle directly involved in the motor task did not show any significant differences between IF and EF. As the target muscle depends on corticospinal drive, we would have expected differences in corticospinal activation due to the altered motor performance. However, MEPs in a single-pulse TMS paradigm are compound muscle action potentials (CMAP) that provide information about the overall excitability of the cortico-spinal tract^29^. For this specific reason, the compound signal evoked by single-pulse TMS is not able to give distinct information about changes in the excitability/inhibition of different contributors such as fast (direct waves) and slower (indirect waves) motor pathways^29^. In order to gain more information about the contribution of fast and slow(er) corticospinal pathways, other methods are required, such as the combination of peripheral nerve stimulation (PNS) and single-pulse TMS with different interstimulus intervals (ISIs). This so-called H-reflex conditioning technique allows to disentangle the excitability of the different slow and fast motor pathways that contribute to the motor command^30–33^. Thus, the H-reflex conditioning technique is an efficient mean to assess the activation but also modulation of the fast and slow(er) motor pathways that contribute to the MEP.

So far, it is unknown whether different motor pathways are sensitive to different attentional strategies. The present study therefore aimed to assess the activity of motor pathways (fast and slower) during a rest condition (REST) and a tonic contraction (comparing an EF and IF) using the H-reflex conditioning technique. Based on previous studies showing that especially the excitability of slow(er) motor pathways is modulated between tasks and conditions and that these pathways are of cortical origin^33–35^, we hypothesized that adopting an altered focus of attention would result in activity changes of the slow(er) but not the direct/fast motor pathways, which were speculated to be essential for the execution of the movement and thus, reduction in these pathways would seem counterproductive. Additionally, based on the ‘constrained action hypothesis’ cited above, we expected to find less activation in the slow(er) motor pathways with an EF contrasted to an IF, because an EF typically results in a more economic activation of neural networks^11,27^.

## Materials and methods

### Participants

Fourteen subjects (25–38 years, 5 women) agreed to participate in this study. All volunteers were right foot dominant, were free from any known orthopaedic or neurological disorders and were naïve to the aim of the study. Participants were only included if they had no education with respect to the effects of an altered attentional focus. They all gave their informed written consent before the experiments, which were approved by the local ethics committee (Commission cantonale d'éthique de la recherche sur l'être humain, CER-VD, Switzerland) and were in accordance with the Declaration of Helsinki.

### Experimental protocol

#### Setup and motor task

**Figure 1:**
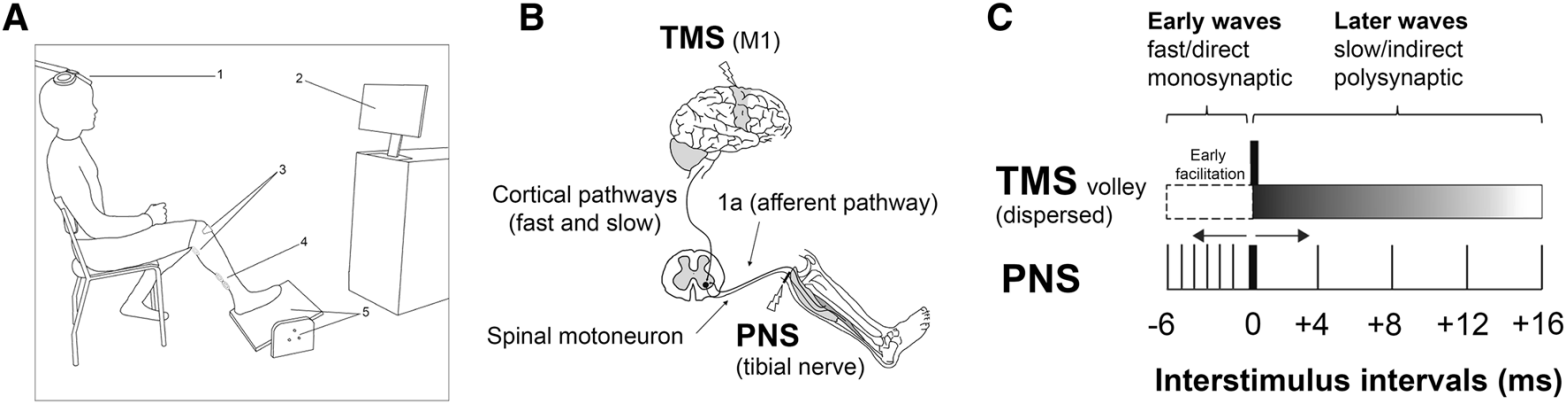
(**A**) Shown is the experimental setup. The TMS coil was placed over the left primary motor cortex (1) as the participants were seated in an upright position facing a monitor placed in front of them (2). In order to evoke H-reflexes in the soleus muscle (SOL), an anode was attached to the anterior aspect of the knee and a cathode was placed in the popliteal fossa (3). To record electrical muscular activity, bipolar surface electrodes were placed on the right SOL (4). Participants were asked to place the right foot on a pedal (5) with a knee angle between 120° and 130°. The position of the pedal which the foot controlled was continuously displayed on the monitor in front of them (2). Participants were asked to match the position of the pedal with the target line displayed on the screen adopting either an external (EF) or an internal focus of attention (IF). The verbal instructions inducing an EF or an IF were given before the motor task. (**B**) The H-reflex conditioning technique allows investigating the excitability of the cortico-spinal tract in further details: it allows differentiating between slow and fast corticospinal motor pathways emerging and projecting from M1. By stimulating the primary motor cortex (M1) with transcranial magnetic stimulation (TMS) and the tibialis nerve with peripheral nerve stimulation (PNS) at the same time, the synaptic input from afferent volleys at the spinal level (H-reflex loop: 1a afferent pathway—spinal motoneuron) will coincide with the descending cortico-spinal volley evoked by TMS. The final responses are conditioned H-reflexes in the SOL muscle, which can be recorded with surface EMG and then compared between conditions in terms of amplitudes. (**C**) The descending volley evoked by TMS over M1 is dispersed. The early waves are attributed to fast corticomotor pathways whereas later waves are attributed to slower pathways. The PNS stimulation can be shifted in order to coincide with different parts of the TMS-induced descending volley. The first response observed in the muscle is called the early facilitation and represents the earliest arriving cortical input evoked by TMS that coincides with the input evoked by PNS. The early facilitation was identified and adjusted for each participant between interstimulus intervals (ISIs) between − 6 and − 1 ms (PNS sent before TMS). All other ISIs (+ 4, + 8, + 12, + 16 ms) were identical for all participants (PNS after TMS).

During the laboratory session, the participants were seated in an upright position in an adjustable chair facing a monitor placed 2.5 m in front of them (see Fig. 1A). They were asked to place the right foot on a pedal with a knee angle between 120° and 130°. The position of the pedal was continuously displayed on the monitor in front of the volunteers and participants were asked to match the position of the pedal with the target line displayed on the screen. The maximal voluntary contraction (MVC) was determined for each participant with an isometric plantar flexion inducing a contraction of the gastrocnemius and soleus muscles. The subjects were then asked to produce 15% of their MVC during the actual test conditions with an EF and an IF.

#### Conditions

While performing the motor task, H-reflex conditioning ISI-curves were first recorded without any focus-specific instructions or muscle contraction (REST condition). This was done to (1) determine the early facilitation (fast motor pathways) individually for each participant (see the ‘Conditioned H-reflex and stimulation timings’ section below for more details) and (2) compare the modulation of the ISI-curve (fast and slower motor pathways) during the REST condition (no contraction, no focus) with both attentional conditions (tonic contraction, EF versus IF). All participants therefore started with the REST condition.

Participants were then either asked to adopt an EF or an IF while ISI-curves were recorded for each condition. The order of these two conditions (EF and IF) was randomized between participants in order to exclude sequence of order effects. The motor task was performed 10 times with an EF and 10 times with an IF. The instructions were given before the execution of the motor task and were formulated as similar as possible. The instruction for the EF condition was ‘Concentrate on the position of the pedal. When the position of the pedal changes, the thickness of the red line displayed on the screen changes. Correct the position of the pedal until the red line is thin again. The line you see on the screen reflects the angle of the goniometer attached to the pedal’. The instruction for the IF condition was ‘Concentrate as hard as possible on the position of your foot. When the position of your foot/ankle changes, the thickness of the red line displayed on the screen changes. Correct the position of your foot/ankle by contracting the muscle of your calf until the red line is thin again. The line you see on the screen reflects the position of your ankle’.

### Apparatus

#### Surface electromyography (EMG)

After skin preparation, bipolar surface electrodes (Blue Sensor P, Ambu A/S®, Ballerup, Denmark) were placed on the muscle belly of the right soleus (SOL). Medially above the tibial bone, a reference electrode was placed and muscular activity was recorded with an inter-electrode distance of 2 cm according to the SENIAM guidelines^36^. EMG recordings were sampled at 4 kHz (PCI-6229, National Instruments, Canyon Park, USA), amplified (× 1000), band-pass filtered (Butterworth 10–1000 Hz). All data was recorded (LabView-based software, Imago Record, Pfitec, Endingen, Germany) and stored on a computer for off-line analysis.

#### Transcranial magnetic stimulation (TMS)

Single-pulse transcranial magnetic stimuli with a monophasic waveform were applied to the left M1 using a MagVenture Pro stimulator (MagVenture A/S, Farum, Denmark) with a 95 mm focal figure-of-eight coil (MagVenture D-B80) to induce MEPs in the SOL. The coil was aligned tangentially to the sagittal plane with its centre 1–2 cm to the left of the vertex and moved stepwise to the left hemisphere to find the optimal position for evoking MEPs in the SOL muscle of the contralateral (right) leg. The handle of the coil was pointing backwards so that a posterior-anterior current in the brain was induced. Once found, the SOL motor hotspot position was recorded and constantly controlled by a neuronavigation system (Polaris Spectra, Northern Digital Inc., Waterloo, Canada and Localite TMS Navigator Version 2.0.5, LOCALITE GmbH, Sankt Augustin, Germany). Then, the active motor threshold (aMT) during the tonic contraction at 15% of MVC was determined to the nearest of 1% of maximal stimulator output and was defined as the minimal stimulation intensity required evoking MEPs bigger or equal to 100 μV peak-to-peak amplitude in 5 out of 10 trials^37^. The stimulation intensity then used during the experiment was set at 90% of aMT during the tonic force-matching task while 90% of resting MT was used for the REST condition (no contraction and no specific focus of attention).

#### Peripheral nerve stimulation

The posterior tibial nerve was electrically stimulated (Digitimer DS7Q, Digitimer Ltd, Hertfordshire, UK) by square-wave pulses of 1 ms duration to evoke H-reflexes in the right SOL. A 5 by 5 cm rubber electrode (anode) was attached to the anterior aspect of the knee. A cathode of 2 cm diameter was moved stepwise in the popliteal fossa in order to find the best position for eliciting H-reflexes in the SOL^38^ (see Fig. 1A, B). After the optimal position was found, the cathode was replaced by a surface EMG electrode and attached to the back of the knee with tape. The time interval between successive stimuli was 5 s and the intensities ranged from 5 to 15 mA. An H-reflex curve^38^ was then recorded while the participant was sitting/resting (no contraction) and sitting while pressing on the pedal (see Fig. 1A). The size (maximal peak-to-peak amplitude) of both M-wave (Mmax) and H-reflex (Hmax) was determined and calculated in Matlab.

#### Conditioned H-reflex and stimulation timings

Compared to TMS alone, H-reflex conditioning of the SOL muscle allows the distinction of different corticospinal projections from M1 during activity and at rest^30–33^ (see Fig. 1B, C). For this purpose, the effect of a subthreshold conditioning stimulus (single-pulse TMS) on a test stimulus (H-reflex at 20% of Mmax) is assessed by varying the inter-stimulus intervals (ISI, see Fig. 1C) between the conditioning TMS pulse and the test H-reflex stimulus^30^. This method allows to investigate the excitability of the different motor pathways (fast vs slower) and provides a more detailed view of the excitability of the corticospinal tract than the analysis of a compound single pulse MEP.

During the REST condition, a conditioning protocol at rest with the following ISIs was tested in a similar way than in a previous study^34^: − 6, − 5, − 4, − 3, − 2, − 1, + 4, + 8, + 12, + 16 ms. Positive ISIs indicate that H-reflexes were evoked after TMS whereas negative ISIs means that PNS was applied prior to TMS (see Fig. 1C).

The latency of the peripheral H-reflex volley to arrive at the moto-neuron pool is 2 to 5 ms longer than the descending volley of the TMS. Thus, the earliest conditioning effect of the corticospinal pulse on the test H-reflex can be detected when the peripheral pulse is evoked 2 to 5 ms before the TMS (ISIs − 2 to − 5 ms). The early facilitation ISI is thought to represent the earliest arriving synaptic input from the descending cortico-spinal volley that coincides with the earliest arriving synaptic input from afferent volleys at the spinal level^30,34,39^. Thus, the early facilitation could be identified from the ISI-curve measured during the REST condition as the ISI that resulted in the first facilitation peak followed by an inhibition of the test H-reflex 1 to 2 ms later. The early facilitation was investigated and identified individually for each participant. In addition, another 4 positive ISIs (+ 4, + 8, + 12 and + 16 ms) were tested in order to reflect the excitability of slower motor pathways (late facilitation or inhibition). If, for example, the early facilitation was identified at ISI − 4 ms in the REST condition, the following ISIs were used in order to compare both conditions (EF vs IF): − 4, + 4, + 8, + 12, + 16 ms. A test H-reflex (which was not conditioned by TMS) and a test MEP (TMS alone) were also included in the experimental protocol. During the REST condition (no contraction and no specific focus instructions), 10 responses for each ISI (conditioned H-reflexes) as well as 10 test H-reflexes and test MEPs were recorded (120 stimulations). Then, 70 responses (1 test MEP, 1 test H-reflex and 5 ISIs * 10 stimulations per ISI) were recorded for each condition (EF vs IF) resulting in another 140 stimulations. The conditions (EF and IF) and the order of ISIs were randomized. The ISI-curve obtained during REST condition was used in the final analysis in order to compare its modulation with both attentional conditions ISI-curves (EF vs IF vs REST). The recording of the REST condition lasted approximately 20 min while the IF and EF conditions took approximately 15 min. The recordings took that long because participants could rest for 30 s after every 12 (REST) or every 7 (IF and EF) stimulations.

### Data processing, analyses and statistics

The raw data acquired was processed and analysed offline using custom MatLab scripts (R2018b, Mathworks, Natick, MA, USA). For all statistical analyses, R version 3.2.4 software (R Foundation for Statistical Computing, Vienna, Austria) was used and the level of significance was set at *P* ≤ 0.05. Unless indicated otherwise, data are reported as mean ± standard deviation. Before the statistical analyses, all results were tested for normal distribution using the Shapiro–Wilk test.

#### Position of the pedal

The goniometer signal was low pass filtered at 50 Hz and transformed in degrees (°). Two separate RMS of the signal subtracted from the target position were computed: over a 3 s window, from 800 to 3800 ms after each trigger (behavioural parameter, to compare the movement accuracy under both attentional conditions). The absolute deviation of the pedal position from the target position was used as the behavioural output parameter. The average of 70 trials in each condition (EF vs. IF) was used for this analysis. A paired Student *t*-test was performed to compare, the accuracy of the task (3 s window) between both conditions.

#### Neurophysiological responses

For conditioned and unconditioned H-reflexes in the SOL, the size (peak-to-peak amplitude) of the responses was evaluated from the unrectified EMG. The test H-reflexes (unconditioned) were considered as a baseline in order to normalise the conditioned H-reflexes. Thus, the size of the intra-individual mean of the conditioned H-reflexes for each ISI was expressed as a percentage to the intra-individual mean of the test H-reflex amplitude of the corresponding condition. Based on these analyses, one ISI-curve per condition (EF, IF and REST) and participant was computed.

A one-way repeated measures ANOVA was performed to compare the effect of ‘condition’ (two levels: EF and IF). In addition, a one-way repeated measures ANOVA was performed to compare the effect of ‘condition’ (three levels: EF, IF and REST) on the early facilitation. Additionally, a two-way repeated measures ANOVA was computed to compare the effect of ‘condition’ (three levels: EF, IF and REST) and the effect of ‘ISI’ (four levels: + 4, + 8, + 12 and + 16 ms). If the sphericity was violated (Mauchly’s test), degrees of freedom were corrected by Greenhouse–Geisser estimates of sphericity. Effect sizes are represented as general eta-squared values^40^ and the Bonferroni procedure was used to correct for multiple comparisons in case of significant *F* values.

The peak-to-peak single-pulse MEPs measured in the SOL muscle were compared between the EF and the IF conditions using a paired Student *t*-test. Finally, the test (unconditioned) H-reflexes measured in the SOL muscle were compared between the EF, IF and REST condition using a one-way ANOVA.

#### EMG levels

EMG levels in the SOL muscle were evaluated by calculating root mean square EMG values (RMS analysis) in a time window of 100 ms preceding the neurophysiological stimulations (H-reflex and/or TMS). The average of 70 trials in each condition (EF and IF) was used in the final analysis. In order to check for differences in background EMG between the two conditions (EF and IF), a paired Student *t*-test was performed.

## Results

### Behavioural output parameter

**Figure 2:**
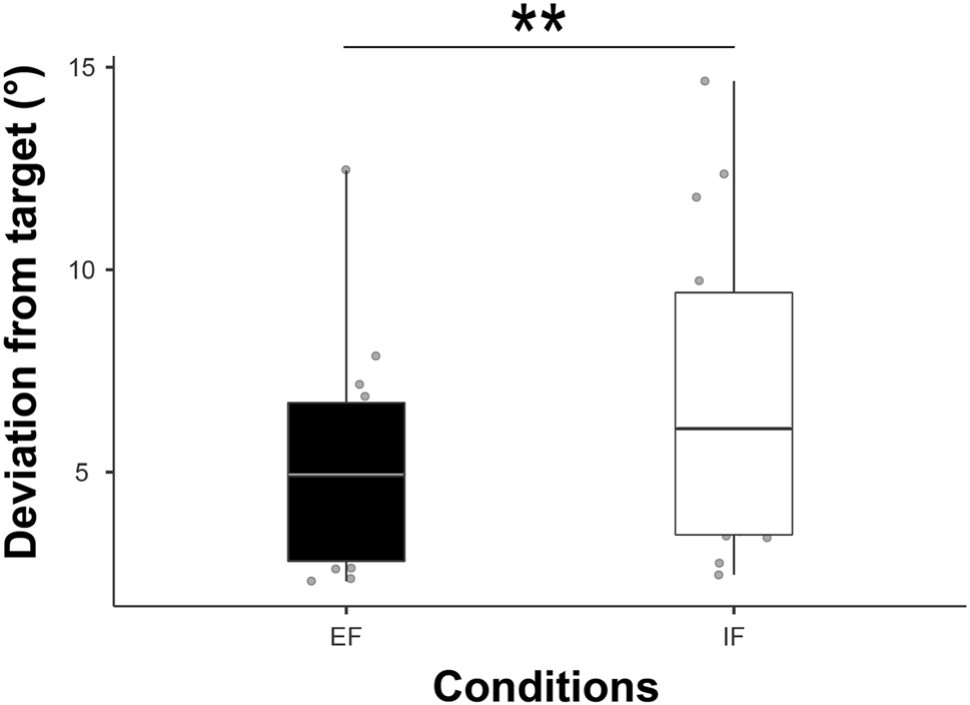
Shown are individual values (*n* = 14) and boxplots comparing both attentional foci (EF and IF) of the deviation from the target position of the pedal in degrees (°). Participants could better match the target line by moving the pedal with their foot when adopting and EF contrasted to an IF (around + 24% improvement). Left boxplot (black) represents the external focus of attention condition (EF) and the right one (white) the internal focus of attention condition (IF). ** *P* ≤ 0.01.

Controlling their right foot on the pedal, participants showed significantly less deviation from the target position (*t*_13_ = − 4.02, *P* = 0.001) when adopting an EF (5.24° ± 2.84°) compared to an IF (6.96° ± 4.03°, see Fig. 2).

### Neurophysiological output parameter

#### Conditioned H-reflexes

**Figure 3:**
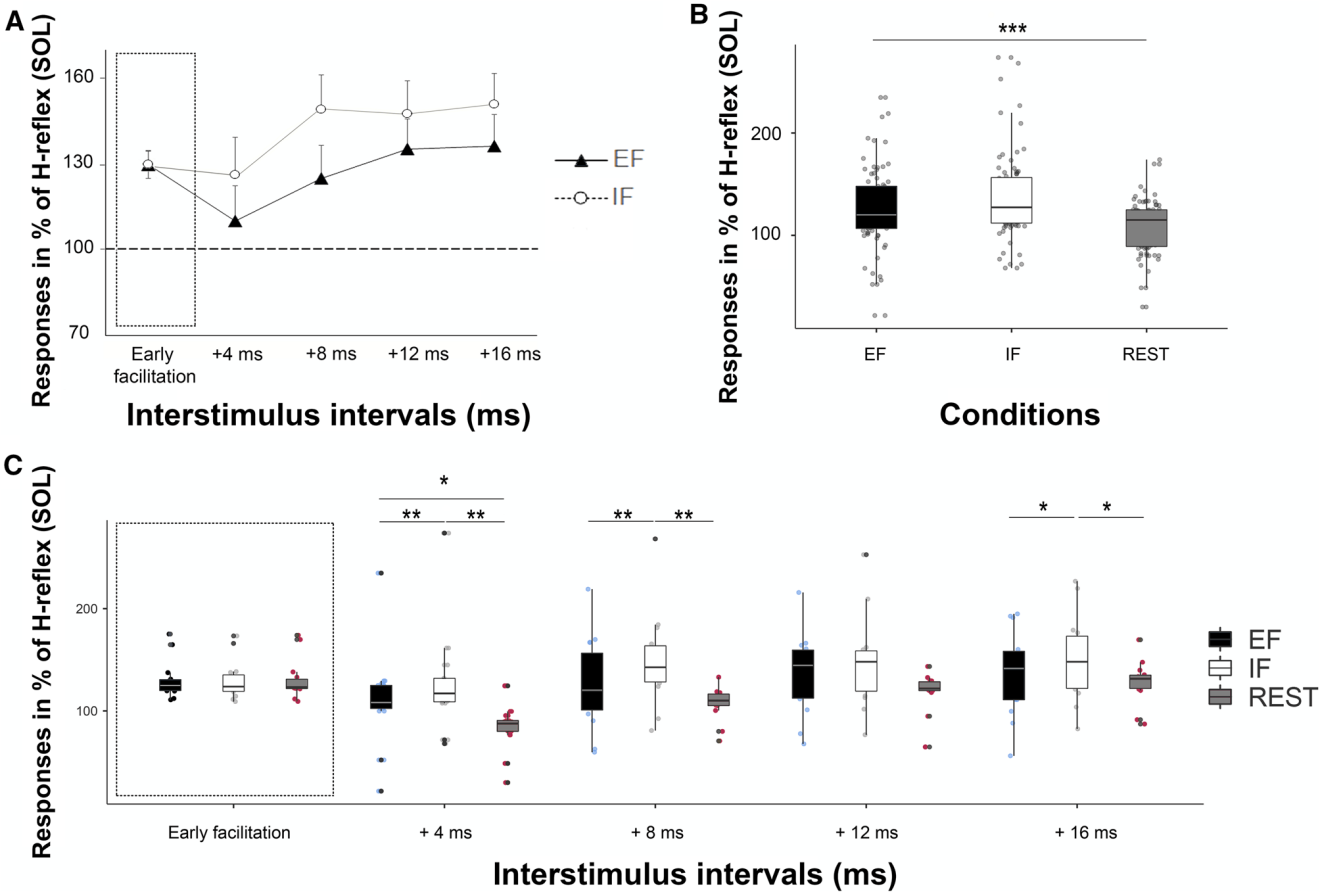
In order to normalise the conditioned H-reflexes in percent (%) for each participant, the unconditioned test H-reflex (not represented) was considered as the baseline. In all graphs, black boxplots/shapes represent the external (EF), the white ones the internal focus of attention (IF) and the grey ones the REST condition (no contraction and no focus). On the abscissa of (**A**) and (**C**), the early facilitation was adapted individually for each participant as the ISI that resulted in the first facilitation peak followed by an inhibition of the test H-reflex 1–2 ms later. (**A**) Shown are mean values and SEM (*n* = 14) of the peak-to-peak amplitudes of the conditioned H-reflexes at five different interstimulus intervals (ISIs : early facilitation, + 4, + 8, + 12 and + 16 ms) under both attentional strategies (EF and IF) in the right soleus muscle. On the abscissa, the ‘early facilitation’ was adapted individually for each participant as the ISI that resulted in the first facilitation peak followed by an inhibition of the test H-reflex 1–2 ms later. When comparing ISI curves, post hoc comparisons (see C for all interactions) showed that adopting an EF resulted in reduced conditioned H-reflexes at ISIs + 4, + 8 ms and + 16 ms, when compared with an IF. (**B**) Shown are individual values and boxplots of the of the peak-to-peak amplitudes of the conditioned H-reflexes comparing the three conditions (EF, IF and REST). The main effect of condition revealed that conditioned H-reflexes at REST were significantly smaller when compared with H-reflexes at EF and IF. *** *P* ≤ 0.001. (**C**) Shown are individual values (*n* = 14) and boxplots of the peak-to-peak amplitudes of the conditioned H-reflexes at five different interstimulus intervals (ISIs : early facilitation, + 4, + 8, + 12 and + 16 ms) under both attentional strategies (conditions: EF and IF) and the REST condition (no contraction and no specific focus of attention) in the right soleus muscle. When comparing the early facilitation, no significant difference was found between the conditions (EF, IF and REST). When comparing the ISI curves between the EF and IF condition, post hoc comparisons showed that adopting an EF resulted in reduced conditioned H-reflexes at ISIs + 4 (around − 16%) + 8 ms (around − 25%) and + 16 ms (around − 10%), when compared with an IF. The H-reflexes measured at the + 4 ms ISI were significantly smaller during the REST condition than the EF and or IF conditions. In order to normalise the conditioned H-reflexes in percent (%) for each participant, the unconditioned test H-reflex (not represented) was considered as the baseline.

When analysing the effect of ‘condition’ on the size of conditioned H-reflexes (see Fig. 3A), the 2 [condition: EF and IF] by 5 [ISIs: early facilitation, + 4, + 8, + 12 and + 16 ms] ANOVA revealed a significant main effect of ‘ISI’ *(F*_2.56, 33.28_ = 2.58, *P* = 0.04, *η*^*2*^ = 0.05) and a significant main effect of ‘condition’ (EF vs. IF, *F*_1, 13_ = 25.67, *P* < 0.001, *η*^*2*^ = 0.03). The ANOVA also revealed a significant interaction effect of ‘ISI’ by ‘condition’ (*F*_2.64, 34.32_ = 4.13, *P* = 0.004, *η*^*2*^ = 0.01).

When analysing the effect of ‘condition’ (EF, IF and REST) on the size of the early facilitation, the one-way ANOVA revealed no significant effect (*F*_2, 26_ = 0.18, *P* = 0.83, *η*^2^ < 0.001). When analysing the effect of ‘condition’ on the size of conditioned H-reflexes (see Fig. 3), the 3 [condition: EF, IF and REST] by 4 [ISIs: + 4, + 8, + 12 and + 16 ms] ANOVA revealed a significant main effect of ‘ISI’ *(F*_1.56, 20.28_ = 10.84, *P* = 0.001, *η*^*2*^ = 0.10) and a significant main effect of ‘condition’ (EF vs. IF vs. REST, *F*_1.18, 15.34_ = 11.41, *P* = 0.002, *η*^*2*^ = 0.12). The ANOVA also revealed a significant interaction effect of ‘ISI’ by ‘condition’ (*F*_6, 78_ = 2.53, *P* = 0.02, *η*^*2*^ = 0.01).

For the main effect of ‘condition’ (see Fig. 3B), pairwise post hoc comparisons revealed that conditioned H-reflexes at REST (10.9.45 ± 25.50%) were significantly smaller when compared with H-reflexes in the EF (126.69 ± 42.91%, *P* = 0.001) and IF (143.52 ± 44.75%, *P* < 0.001) conditions. Additionally, H-reflexes in the EF condition were significantly smaller when compared with H-reflexes in the IF condition (*P* < 0.001).

Pairwise post hoc comparisons for the interaction effect of ‘ISI’ by ‘condition’ (see Fig. 3C) revealed that conditioned H-reflexes in the EF condition at ISI + 4 ms (110.03 ± 46.24%) were significantly smaller than in the IF condition (126.28 ± 48.94%, *P* = 0.002). In addition, H-reflexes at ISI + 4 ms in the REST condition (83.09 ± 22.17%) were significantly smaller than in the EF (*P* = 0.03) and IF (*P* = 0.006) conditions. In addition, pairwise post hoc comparisons revealed that conditioned H-reflexes in the EF condition at ISI + 8 ms (124.95 ± 43.64%) and + 16 ms (136.40 ± 41.42%) were significantly smaller than in the IF condition (+ 8 ms : 149.17 ± 45.17%, *P* = 0.004 and + 16 ms : 150.99 ± 40.96%, *P* = 0.04). Additionally, H-reflexes in the REST condition at ISI + 8 ms (107.34 ± 15.73%) and + 16 ms (128.23 ± 20.59%) were significantly smaller than in the IF condition (+ 8 ms : *P* = 0.005 and + 16 ms : *P* = 0.03 ) but not than the EF condition (+ 8 ms : *P* = 0.26, + 16 ms : *P* = 0.87). Finally, the size of the H-reflexes at ISI + 12 did not differ significantly between conditions (EF : 135.37 ± 39.33%, IF : 147.66 ± 43.89% and REST : 119.14 ± 18.86%, all *P* > 0.05). However, as it can be seen in Fig. 3A, C, the ISI + 12 is not an outlier but follows pretty well the other (significant) results. Nonetheless, due to multiple comparisons and extensive corrections of the *p*-value, the *p*-value at ISI + 12 did not reach the level of significance^41^. However, the uncorrected value of ISI + 12 comparing both attentional conditions (EF vs IF) is *p* = 0.04. Thus, we do not think that ISI + 12 is any different from the other positive ISI’s.

#### Control output parameters

**Figure 4:**
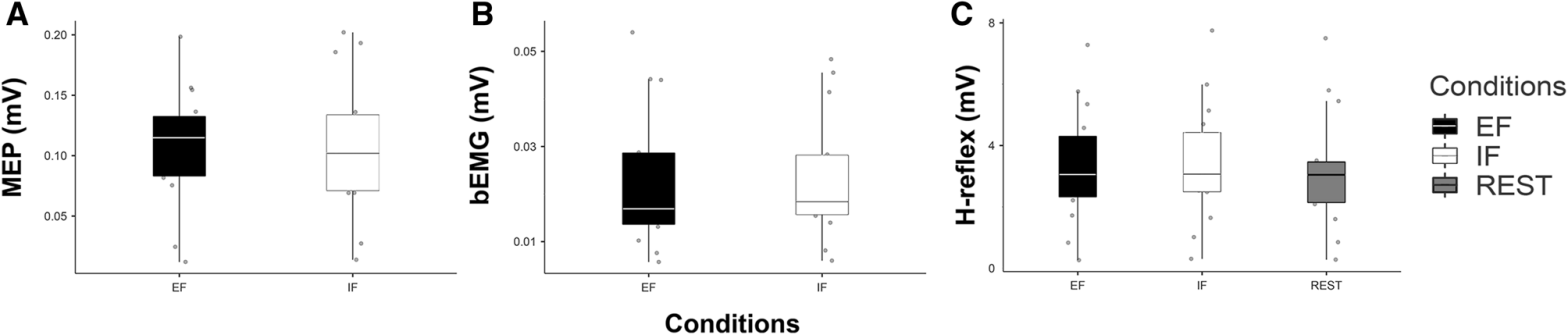
In all graphs, shown are individual values (*n* = 14) and boxplots comparing of (**A**) the test single-pulse MEPs (unconditioned) under both attentional strategies (EF and IF), (**B**) background activity of the SOL muscle 100 ms before TMS and/or PNS (bEMG) under both attentional strategies (EF and IF) and (**C**) the test H-reflexes (unconditioned) under both attentional strategies and the REST condition (no contraction and no specific focus of attention). No significant difference was found in all these parameters between conditions (all P > 0.05 In all graphs, black boxplots represent the external (EF), the white ones the internal focus of attention (IF) and the grey ones the REST condition (no contraction and no focus).

When analysing test MEP peak-to-peak amplitudes (single-pulse TMS alone) in the SOL muscle (see Fig. 4A), no significant difference (*t*_13_ = − 0.09, *P* > 0.05) between attentional conditions was found (EF = 0.106 ± 0.049 mV and IF = 0.107 ± 0.057 mV). In addition, when comparing the amount of muscle activity (bEMG) in the 100 ms before stimulation (TMS and PNS) in the SOL muscle, no difference between attentional conditions was found (*t*_13_ = − 0.45, *P* > 0.05, see Fig. 4B) indicating that background activation did not have an impact of the neurophysiological data. Finally, when analysing test H-reflex peak-to-peak amplitudes (unconditioned) in the SOL muscle, no significant difference (F_*1.22, 15.86*_ = 1.34, *P* = 0.27, *η*^*2*^ = 0.001) between conditions was found (EF = 3.30 ± 1.91 mV, IF = 3.39 ± 1.97 mV and REST = 3.20 ± 1.94 mV, see Fig. 4C).

## Discussion

For the first time, the excitability of distinct fast and slower motor pathways under different attentional conditions (EF vs IF) was investigated. The main finding of the present study was that distinct neuron populations within M1 that are responsible for the indirect and slower projections result in a focus-specific modulation. More specifically, adopting an EF (versus an IF) reduced the activity of slow(er) motor pathways but faster corticospinal pathways seemed not affected by the different attentional foci. Moreover, when focusing externally (EF), participants displayed better motor performance.

### Attentional foci and motor behaviour

In the present study, the comparison of the motor task accuracy between both attentional strategies was conducted to confirm that motor performance can be improved when adopting an EF compared with an IF (see Fig. 2). As expected and as reported by multiple previous studies (see^14^ for a detailed review), motor performance was increased when using an EF compared with an IF. When focusing externally, participants showed greater accuracy (less deviation) when controlling the pedal in order to match its position with the target one. These results confirm previous observations^9,42^, indicating more accuracy during different motor tasks with an EF contrasted with an IF. Thus, from a behavioural point of view, our results are well in line with previous studies indicating enhanced motor performances when adopting an EF contrasted with an IF^14^.

### Attentional foci and neurophysiological changes

When looking at the overall spinal excitability (H-reflexes alone, not conditioned, see Fig. 4C) and the overall cortico-spinal excitability (control single-pulse MEPs, see Fig. 4A), no significant difference between both conditions (EF versus IF) was found in the present study. These results are in line with previous works^11,27^, indicating no significant difference in single-pulse MEP amplitudes measured in the prime mover (agonist muscle, directly involved in the motor task). However, this so-called compound potential elicited by single-pulse TMS over M1 can only give information about the overall excitability of the cortico-spinal tract^29^ but can neither differentiate between excitatory and inhibitory influences (see^11,27^) nor between the activity of fast and slower cortico-motor pathways. Therefore, the current study used the H-reflex conditioning technique to differentiate activity in fast (direct) and slower (indirect) motor pathways with an attentional strategy (EF vs IF) and during the REST condition (no contraction). In line with previous research^33–35^, we show that tonic contractions (during both attentional strategies EF and IF) result in an increased activity of the slow(er) motor pathways compared to the REST condition (no contraction).

More importantly, we show for the first time that altering the attentional focus results in changes of the activity of slow(er) motor pathways (polysynaptic pathways) but not of fast (early facilitation: monosynaptic pathways) motor projections. The diminished activation of these slower pathways being accompanied by an enhanced motor performance (less deviation from the target line) with an EF provides further evidence for a more economic neural strategy when an EF is adopted. Our data therefore support, from a neural perspective, the ‘constrained action hypothesis’ postulated in the early 2000s^12,13^ stipulating that improved motor control with an EF is due to a more efficient mode of motor control that involves fast and unconscious control processes (see “Supporting the “constrained action hypothesis” from a neural perspective” section below for details).

Previous studies demonstrated that the increased movement efficiency usually associated with an EF might be due to differential neural activation, such as enhanced motor surround inhibition (SI)^11^. Shaping neuronal activity in the motor system by suppressing excitability in the areas surrounding an activated neural network^43,44^, the SI mechanism can be considered as a strategy to improve the spatial restriction of the motor command. Thus, the increased surround inhibition associated with an EF found in Kuhn and colleagues’ study^11^ might be considered as an improved spatial focus. On the other side, the decrease in excitability of slower motor pathways when adopting an EF may be considered as an improved temporal focus of the motor command. The temporal restriction may help to better understand the improved motor performance usually associated with an EF. Moreover, together with the enhanced intracortical inhibition^27,28^ and the increased SI^11^, the down-regulated activation of slower motor pathways found in this study might be considered as a supplementary neural strategy to better focus the cortical motor command, likely being one reason for the beneficial behavioural effects when adopting an EF. Finally, both of these strategies (temporal and spatial restriction) would nicely help to better understand the improved movement efficiency usually associated with an EF, by indicating a more economic mode of neurophysiological control.

### Supporting the “constrained action hypothesis” from a neural perspective

To explain the beneficial effects of using an EF over an IF-strategy, the constrained action hypothesis was postulated^12,45^. However, this psychological theory was based almost exclusively on behavioural studies and thus, supported the positive effects of an EF from a broad perspective. Over almost 20 years (and still today), this theory has been used to support the enhanced performance and more efficient motor control usually associated with an EF over an IF (see^14^ for a detailed review). However, studies supporting it from a neurophysiological perspective were missing for many years. The first physiological measures in this field of research were done with EMG and showed reduced muscular activation despite enhanced motor performances when adopting an EF compared to an IF^5–11^. In contrast to these reduced activation levels in muscles, an enhanced neural activation was reported for cortical neurons in fMRI studies^20,21,46–48^. The reason for the enhanced brain activation with an EF could not be well interpreted, because a more efficient motor control is often associated with less brain activation^49,50^. However, the BOLD signal measured with fMRI is a compound potential that accumulates the activation levels of both inhibitory and facilitatory neurons^23^. Therefore, the possibility exists that one measures an enhanced BOLD signal because of an enhanced activation in inhibitory neurons that reduce the output of upper motoneurons. In fact, recent TMS studies showed that better motor performances was accompanied by enhanced levels of cortical inhibition in the prime mover^27,28^ but also the surrounding muscles^11^. Thus, adopting an EF seems to result in an altered cortical activation resulting in more inhibition that consequently reduces activation in upper motoneurons. The present study adds another puzzle piece to this line of argumentation since the activation of slow(er) motor pathways is also reduced with an EF compared to an IF. We do not know if the ‘over-activation’ of the slow(er) motor pathways in the present study with an IF are directly linked to reduced inhibitory processes, but the data show that adopting an IF leads to enhanced activation of slow(er) motor pathways that are not needed for an efficient motor execution. Thus, the current study nicely complements previous studies^11,27,28^ supporting the constrained action hypothesis by showing an improved and more efficient neural strategy when activating the muscles with an EF.

### Limitations and future directions

It was demonstrated in this study that slower motor pathways activating lower limb muscles are sensitive to different attentional strategies. However, the precise neurophysiological origin of the slower motor pathways is not fully understood. Although it is known that the effects mediated via slower motor pathways hinge on the activity of cortical neurons, suggesting a cortical origin^33^, it seems that further studies are needed to explore the exact anatomical pathways of the slow(er) motor pathways. Thus, our findings are able to shed light on the modulation of slower motor pathways, but cannot provide more information about network-related mechanisms of the brain. It is further not clear, whether the reduction in the activity of slow(er) motor cortical pathways is directly related to changes in inhibitory motor cortical circuits. In addition, we do not know if the results found for lower limb muscles can be generalized to upper limb muscles.

The present study outlined the immediate effects of adopting diverse attentional strategies on motor performance and on activity parameters of different motor pathways. Although we think that our results are meaningful and essential, it would also be relevant to explore the effect of learning and/or practicing with different foci of attention on brain activity in the long term. This could be conducted using the same TMS protocols as in previous studies^11,27,28^ and including this one, but also by adding new techniques such as the proton magnetic resonance spectroscopy (MRS) and/or by exploring the connectivity of brain motor networks with fMRI^51^.

## Conclusion

The results of the present study shed further light on the neural correlates underlying attentional foci and help to better understand the positive mechanisms of an EF on different motor pathways (fast and slow) in the healthy motor system. The highlight of the present study is that directing attention externally leads not only to an improved motor performance (more accuracy in the task), but is also accompanied by a deactivation of the slower motor pathways. Together with the cortical modulations (i.e. increased SI and increased intracortical inhibition, see Kuhn and co-workers’ studies^11,27^), it might therefore be argued that the decreased activity of slower (and indirect) descending pathways could be another complementary temporal neural strategy justifying the increased movement efficiency commonly found with an EF compared with an IF.

## Data Availability

On reasonable request, the datasets generated during the present study are available from the corresponding author.
